# Vitamin D Levels and Monospot Tests in Military Personnel with Acute Pharyngitis: A Retrospective Chart Review

**DOI:** 10.1371/journal.pone.0101180

**Published:** 2014-07-10

**Authors:** Sean R. Maloney, David Almarines, Paula Goolkasian

**Affiliations:** 1 Navy Mobilization Processing Site, Deployment Processing Command-East, Camp Lejeune, North Carolina, United States of America; 2 W.G. (Bill) Hefner VA Medical Center, Salisbury, North Carolina, United States of America; 3 Department of Psychology, University of North Carolina at Charlotte, Charlotte, North Carolina, United States of America; University of Oxford, United Kingdom

## Abstract

Some recent studies have proposed an important role for vitamin D in reducing the risk of infection by assisting in the suppression of viruses and by controlling the inflammatory response. A low vitamin D state may have a detrimental effect on the immune system’s ability to produce activated CD8+ T cells, and it may increase the inflammatory reaction to Epstein Barr virus. The aim of this chart review was to see if serum 25 OH vitamin D_3_ levels in service members with acute pharyngitis were lower in those who had positive rather than negative monospot tests. A retrospective chart review was conducted on the medical records of service members who presented to sick call at Camp Lejeune, NC with acute pharyngitis from October 8, 2010 until June 30, 2011. Serum 25 OH vitamin D_3_ levels were compared between those with positive and negative monospot test results. Of the 25 records that were reviewed, there were 9 (36%) service members with positive results, and they were found to have lower vitamin D levels (Median = 20.80 ng/ml, Interquartile range = 10.15) than those with negative test results (Median = 30.35 ng/ml, Interquartile range = 17.05), Mann-Whitney U = 41, *p* = .039. Only 1 of the 9 with positive test results had a normal serum 25 OH vitamin D_3_ level (30 ng/ml or greater) compared with 9 of the 16 with negative test results. Optimal vitamin D stores may play a significant role in reducing the risk of developing acute mononucleosis but larger, prospective studies will be needed to verify these findings.

## Introduction

Acute mononucleosis is a clinical condition that occurs at relatively high frequency in military personnel and college students (11–48 cases per 1000 persons) [1]–[3]. It is most often caused by the Epstein-Barr virus (human herpes virus 4), which is composed of a double helix of DNA wrapped in a protein capsid and surrounded by a protein tegument and a lipid envelope. Acute mononucleosis from Epstein-Barr virus is usually transmitted through saliva and initially infects epithelial cells and B lymphocytes of the acquired immune system where it can become latent [4]. The monospot test (latex agglutination assay using horse RBC’s) is nearly 100% specific for acute mononucleosis in otherwise healthy individuals presenting with acute Pharyngitis [5]. Positive monospot tests can also be associated with leukemia, lymphoma (including Burkitt’s lymphoma), pancreatic cancer, rheumatoid arthritis, serum sickness, and systemic lupus erythematosus [5], [6].

In acute mononucleosis, monospot tests are usually positive within 1 week of the onset of symptoms reaching a peak at 2–5 weeks and then dropping off over 3–12 months [6]. During week 1, after symptoms begin, there can be a 25% false negative rate. During week 2, there can be a 5–10% false negative rate. During week 3, there can be a 5% false negative rate [6]. This in part explains the monospot test’s sensitivity rate of 85% [5]. One aim of this retrospective chart review is to look for evidence that low vitamin D states may make the development of acute mononucleosis from initial infection with Epstein-Barr virus or from reactivation of latent Epstein-Barr virus more likely.

During the last several years, the importance of vitamin D in innate and adaptive immune system function has become better understood. Vitamin D activating (25-hydroxylase and 1 alpha –hydroxylase) enzymes and vitamin D metabolizing (24-hydroxylase) enzyme are present in monocytes/macrophages and mature dendritic cells of the innate immune system and T-helper cells and natural killer cells of the adaptive immune system [7]–[11]. The presence and regulation of these enzymes within immune system cells demonstrate the autocrine, intracrine, and paracrine roles of vitamin D_3_ within the immune system.

Increased intracellular conversion of 25(OH) vitamin D_3_ to 1,25(OH)_2_ vitamin D_3_ stimulates the production of cathelicidin in monocytes/macrophages of the innate immune system. Human cathelicidin is a peptide that causes the destruction of infectious agents [8], [11]. Cathelicidin has been demonstrated to have direct antiviral activity against adenovirus and herpes simplex virus in vitro [12]. Cell culture experiments suggest that vitamin D has significant anti-viral effects against enveloped viruses (including herpetic viruses) [13]. In contrast, increased extracellular 1,25(OH)_2_D_3_ levels (active vitamin D produced by immune system cells) provide negative feedback to activated B and T lymphocytes. This negative feedback limits the proliferation of B lymphocytes, IgG, T lymphocytes and associated inflammatory cytokines of the adaptive immune system [7], [14].

A low vitamin D state may decrease the immune system’s ability to produce activated CD8+ T lymphocytes which attack Epstein-Barr virus infected B lymphocytes. A low vitamin D state may also increase the inflammatory response by CD8+ T lymphocytes in the presence of Epstein-Barr virus infected cells [15]. In young individuals (less than 26 years of age), serum 25 OH vitamin D_3_ levels correlated inversely to antibody reactivity against Epstein-Barr nuclear antigen 1 [16]. Vitamin D supplementation in individuals wintering over in Antarctica has been shown to mitigate Epstein-Barr virus reactivation [17]. To determine if mononucleosis is associated with a low vitamin D state, we reviewed the charts of military patients with acute pharyngitis and compared serum 25 OH levels between those with positive and negative monospot test results.

## Methods

A retrospective chart review was conducted on the medical records of 25 otherwise healthy active duty service members (19 U.S. Marines and 6 Navy Sailors) who presented to sick call with acute pharyngitis between 8 October 2010 and 30 June 2011. There were 7 women in the sample. The service members were evaluated and treated by one reserve navy medical officer who was mobilized to the medical unit of the Navy Mobilization Processing Site, Deployment Processing Command – East, Camp Lejeune, N.C. in September of 2010. This chart review was approved by the Human Use Review Committee and by the Research Committee at the Naval Medical Center, Portsmouth, Virginia. Patient records were anonymized and de-identified prior to data analysis.

In thirteen of the 25 cases reviewed, serum 25 OH vitamin D_3_ levels were drawn on the same day as the monospot test, and in seven vitamin D_3_ levels were drawn within 6 days. The remaining 5 tests were drawn within 7 to 108 days of the monospot test. (Overall range 0 to 108 days, Mean = 13.7 days). No service members were excluded who presented to sick call with acute pharyngitis and who had a serum 25 OH vitamin D_3_ level and a monospot test drawn during the dates of the chart review. Serum 25 OH vitamin D_3_ levels were compared between those with positive and negative monospot test results with a one tailed Mann Whitney U test at the.05 significance level. A nonparametric test was used because of the small sample size and the non normal distribution of the vitamin D levels. The one-tailed test was justified based on the prediction that lower serum 25 OH vitamin D_3_ levels would be associated more prevalently with positive monospot test results in comparison to higher serum 25 OH vitamin D_3_ levels. Low vitamin D states adversely affect immune system function. Statistical analyses were performed using SPSS Statistics Version 19 (IBM Corporation, Armonk, New York).

Serum specimens to be analyzed for 25 OH vitamin D_3_ were evaluated by outside reference labs (Lab Corp of America and Quest Lab). Serum 25 OH vitamin D_3_ reference ranges used by both reference labs were as follows: vitamin D deficiency (<20 ng/ml), vitamin D insufficiency (20 ng/ml to <30 ng/ml), and normal (30 ng/ml to 100 ng/ml) [18]. The serum specimens were analyzed for serum 25 OH vitamin D_3_ at Lab Corp of America by immunochemiluminometric assay performed on the DeaSorin LIASON^R^ instrument and at Quest Diagnostic Nichols Institute Lab by liquid chromatography/Tandem Mass Spectroscopy (LC/MS/MS).

Location in the world and seasonal variation during the year can significantly affect serum 25 OH vitamin D_3_ levels [16]. Because seasonal variation and clustering of cases might affect conclusions reached from this chart review, monospot test results and serum 25 OH vitamin D_3_ levels were also tabulated by month of patient presentation with acute pharyngitis. Serum 25 OH vitamin D_3_ levels and monospot test results were also tabulated to include the dates serum 25 OH vitamin D_3_ levels and monospot tests were drawn, the reference lab used to process the serum 25 OH vitamin D_3_ levels, and the age and sex of the service members. Serum 25 OH vitamin D_3_ levels were generally drawn at the time of acute pharyngitis and were not seasonally adjusted. (See discussion section.) Data for confounding factors of skin pigmentation and race were not available from the medical record at the time of the chart review.

## Results

**Figure 1:**
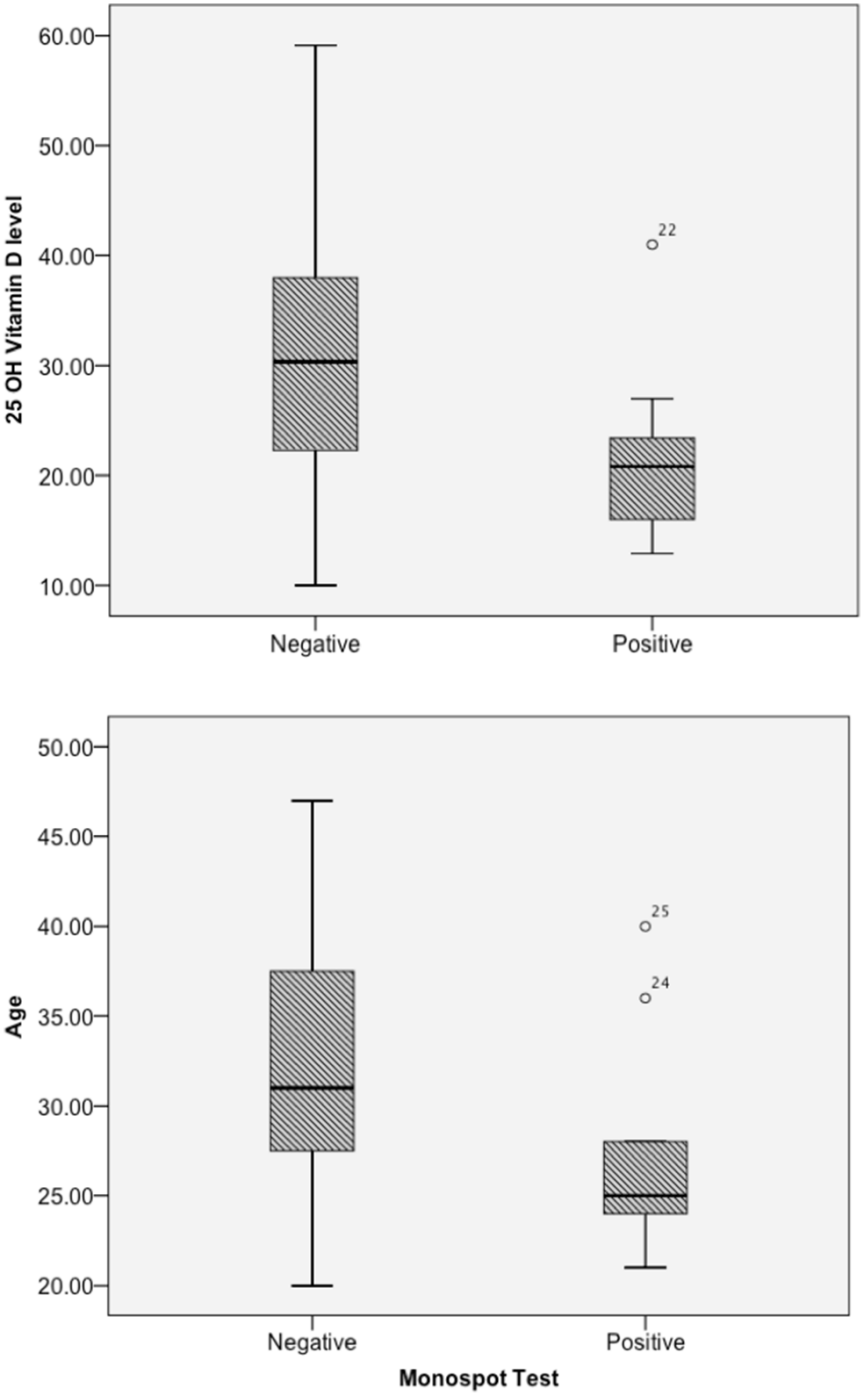
Boxplots comparing Serum 25 OH vitamin D_3_ levels (upper panel) and age (lower panel) between those with acute pharyngitis who test positive and negative on the monospot test.

Among the 25 service members evaluated for acute pharyngitis, monospot tests were positive in 9 (4 women and 5 men). Table 1 compares the groups with positive and negative monospot test results on all of the relevant variables and presents the results of the statistical tests. It is evident from the box plots that are presented in the upper panel of Figure 1, that there are differences between the positive and negative monospot groups in the distribution of serium 25 OH vitamin D_3_ levels. Those with positive test results were found to have distributions that were skewed toward lower vitamin D levels (Median = 20.80, Interquartile Range = 10.15) than those with negative test results (Median = 30.35, Interquartile Range = 17.05). The results of the one-tailed Mann Whitney U test (U = 41, *p* = .039) show that the group differences are significant. Moreover, of the service members with acute pharyngitis and a positive monospot test, only 1 out of 9 had a normal level of serum 25 OH vitamin D_3_ as compared to 9 out of the 16 with a negative monospot test.

**Table 1:** A comparison of group data for those with positive and negative monospot test results.

	Negative Group	Positive Group	Statistical test	*p* level
Sample Size	16	9		
# of females	3	4		
# of males	13	5	Chi Square (1) = 1.86	.17
Vitamin D Data				
Median	30.35 ng/ml	20.80 ng/ml	Mann-Whitney U = 41	.039^1^
IQR^2^	17.05	10.15		
Skewedness	.52	1.58		
STDerror^3^	.56	.72		
Kurtosis	1.36	3.05		
STD error	1.09	1.40		
Age				
Median	31 years	25 years	Mann-Whitney U = 42.5	.094^4^
IQR	10.5	8.50		
Skewedness	.39	1.29		
STDerror	.56	.72		
Kurtosis	.07	.66		
STD error	1.09	1.40		

1 one-tailed test.

2 IQR = Interquartile Range.

3 STD error = Standard error.

4 two-tailed test.

Monospot test results and serum 25 OH vitamin D levels are organized in Table 2 by month that the service member presented to sick call and had their monospot blood test drawn. Cases of acute pharyngitis suspected of having mononucleosis were most frequently seen in during the winter months. There were 9 positive monospot test results. Four were drawn during the fall, four during the winter and one in summer. A complete listing of serum 25 OH vitamin D_3_ levels (lowest to highest) and monospot test results are presented in Table 3 with the dates that tests were drawn, the reference labs that ran the serum 25 OH vitamin D_3_ levels, and demographic information.

**Table 2:** Initial Serum 25 OH Vitamin D_3_ Levels (ng/ml) Drawn from each Service Member and Tabulated by Month of Presentation with Acute Pharyngitis.

Group	Oct.	Nov.	Dec.	Jan.	Feb.	March	April	May	June
PositiveMonospot	12.914.120.8		21.2	27.0	16.018.023.4				41.0
NegativeMonospot	19.659.1	28.931.2	29.030.7	25.038.0	14.038.0	15.030.031.0	10.039.0	38.0	

(The number of entries in each cell signifies the number of monospot tests drawn during that month).

**Table 3:** Serum 25 OH Vitamin D_3_ Levels (ng/ml – lowest to highest) and Monospot Test Results.

25 OH Vit. D_3_ Level	Date _VitD drawn_	Monospot Test	Date _Monospot drawn_	Lab Used	Age/Years	Sex
10.0	6 Apr ‘11	NEG	5 Apr ‘11	Quest	34	M
12.9	21 Oct ‘10	POS	21 Oct ‘10	Lab Corp.	36	F
14.0	17 Feb ‘11	NEG	17 Feb ‘11	Quest	29	M
14.1	18 Oct ‘10	POS	18 Oct ‘10	Lab Corp.	23	M
15.0	17 Mar ‘11	NEG	17 Mar ‘11	Quest	20	M
16.0	15 Feb ‘11	POS	15 Feb ‘11	Quest	40	M
18.0	11 Feb ‘11	POS	11 Feb ‘11	Quest	24	F
19.6	18 Oct ‘10	NEG	13 Oct ’10	Lab Corp.	32	M
20.8	07 Sep ‘10	POS	8 Oct ‘10	Lab Corp.	24	F
21.2	10 Dec ‘10	POS	8 Dec ‘10	Lab Corp.	21	F
23.4	14 Oct ‘10	POS	2 Feb ‘11	Lab Corp.	25	M
25.0	21 Jan ‘11	NEG	21 Jan ‘11	Lab Corp.	38	M
27.0	31 Jan ‘11	POS	26 Jan ‘11	Quest	28	M
28.9	7 Jan ‘11	NEG	3 Nov ‘10	Lab Corp.	40	M
29.0	20 Dec ‘10	NEG	14 Dec ‘10	Lab Corp.	47	M
30.0	25 Mar ‘11	NEG	25 Mar ‘11	Quest	24	M
30.7	14 Dec ‘10	NEG	14 Dec ‘10	Lab Corp.	37	M
31.0	14 Mar ‘11	NEG	14 Mar ‘11	Quest	38	M
31.2	2 Nov ‘10	NEG	2 Nov ‘10	Lab Corp.	28	M
38.0	19 May ‘11	NEG	15 Feb ‘11	Quest	24	M
38.0	10 May ‘11	NEG	5 May ‘11	Quest	27	F
38.0	20 Jan ’11	NEG	20 Jan ‘11	Lab Corp.	30	F
39.0	27 Apr ‘11	NEG	27 Apr ‘11	Quest	29	M
41.0	6 Jul ‘11	POS	30 Jun ‘11	Quest	27	M
59.1	4 Oct ‘10	NEG	4 Oct ‘10	Lab Corp.	33	F

Box plots of the age distributions in the two groups are also shown in the lower panel of Figure 1. The positive monospot group is skewed toward the lower values with a median age of 25 years. However, even though the interquartile range is narrower and the median is lower than the negative group, the Mann-Whitney U test shows that the difference are not large enough for significance with a two-tailed test, (U = 42.4, p = .094).

## Discussion

Lower vitamin D levels in those with acute pharyngitis and a positive monospot test when compared to those with acute pharyngitis and a negative monospot test provides support for a relationship between vitamin D status and acute mononucleosis. This finding is consistent with previous work that shows vitamin D supplementation during an Antartic winter can mitigate Epstein-Barr virus reactivation [17]; and adds to growing evidence that vitamin D plays an important role in the suppression of infectious agents including herpetic viruses by both the innate and adaptive immune systems. Although not significant, the additional finding that the positive monospot group had a lower median age than those with negative test results is consistent with historical data, which demonstrates that young adults in general are susceptible to developing acute mononucleosis in comparison to older adults [1].

Two follow-up questions that arise from these results are whether lower serum 25 OH vitamin D_3_ levels make individuals more susceptible to infection by viruses (such as Epstein-Barr) and whether chronic lower serum 25 OH vitamin D_3_ levels contribute to stronger or perhaps longer accompanying immune inflammatory responses. Comparison of serum 25 OH vitamin D_3_ levels with immune system tests, such as a monospot test or more quantitative specific immunoglobulin tests, may provide a direct way to study relationships which may exist between serum 25 OH vitamin D_3_ levels and immune system function. There are many factors that influence vitamin D status in humans. In the absence of vitamin D nutritional supplementation, one important factor in acquiring vitamin D is the amount of ultra-violate B (UVB) light which reaches the basal layer of the skin to initiate the first step in the synthesis of active vitamin D. The production of vitamin D depends on many geographical features including location with respect to the equator (seasonal variation), altitude, and atmospheric conditions. Several studies have proposed a cosinor model based on the sinusoidal fluctuation in UVB light using data from a large, community-based population with racial diversity. The small number of data points in our study and the lack of sufficient serum 25 OH vitamin D_3_ data from a similar diverse local community of active duty service members precluded the use of a cosinor model to adjust our data for seasonal variation [19]–[21]. In addition, serum 25 OH vitamin D levels at the time of an acute infection may be more reflective of the vitamin D effect on the immune system than a seasonally adjusted vitamin D level.

The current definitions of vitamin D deficiency, insufficiency and normalcy are based on vitamin D requirements for optimal bone health [18]. Vitamin D requirements/levels for optimal immune system health may be different. Larger studies will be needed to determine whether lower vitamin D levels significantly increase the risk of developing acute mononucleosis, increase the severity of the disease, or prolong recovery from it. If low vitamin D levels correlate with any of the above, acute mononucleosis and its relation to vitamin D levels might be used as a model to help determine the optimal serum 25 OH vitamin D_3_ level for immune system function in acute mononucleosis and possibly in other infectious diseases.

Service members with acute pharygitis and vitamin D deficiency or insufficiency (8 of 9 service members with a positive monospot test and 7 of the 16 service members with a negative monospot test) were given vitamin D supplementation as part of their treatment. Recent basic research concerning the paracrine, intracrine, and autocrine functions of vitamin D in immune system function and the low levels of serum vitamin D levels found in this small group of service members with acute phayngitis suggest that checking a serum 25 OH vitamin D_3_ level may be clinically useful in those patients presenting with acute pharyngitis and especially in those presenting with acute pharyngitis and a positive monospot test. Correcting vitamin D deficiency and insufficiency might improve recovery especially from those viruses (i.e. Epstein Barr virus and cytomegalovirus), which become latent and linger in the body.

Conclusions from this chart review are limited by the uncontrolled retrospective nature of the chart review and the small number of charts reviewed. Although age and sex were measured, the groups were not matched on important variables such as race and month of sampling. Also, two different methods were used to measure vitamin D levels and the critical finding of a relationship between the monospot test results and vitamin D levels depended upon a one-tailed rather than a two-tailed statistical test.

Since there can be a 1–3 week delay between the onset of the symptoms of acute pharyngitis due to mononucleosis and a monospot test becoming positive, some of the negative monospot test results might have been positive if drawn at a later date. Military service members at Camp Lejeune, N.C. have very quick access to health care through daily unit sick call. Their evaluations might occur sooner than their civilian counterparts resulting in a higher false negative monospot test rate. The data from this chart review is suggestive of a possible role of lower serum 25 OH vitamin D_3_ levels in the development of acute mononucleosis but larger, controlled, prospective studies will be needed to verify the relationship.

## Acknowledgements and Disclosures

This paper is based on research supported by the U.S. Navy and the Office of Research and Training at Naval Hospital Camp Lejeune during the mobilization of CAPT Sean R. Maloney MC USN as a medical officer to the Navy Mobilization Processing Site, Deployment Processing Command – East, Camp Lejeune, N.C. including part time assignment to the Sports Medicine Clinic, Naval Hospital Camp Lejeune between August 2010 and May 2012.

The welfare of human subjects was protected and the Naval Medical Center, Portsmouth, Institutional Review Board and Research Committee approved all U.S. Navy research involving human subjects. (Study: NHCL.2012.0007 – “Retrospective Record Review of Serum 25(OH)D_3_ Levels in US Marines and Navy Sailors”- Completed 4 June 2012) Portions of this paper were contained in the presentation “Vitamin D Deficiency in US Marines and Navy Sailors at Camp Lejeune, NC”, AMSUS (The Society of Federal Health Professionals) – 118^th^ Annual Continuing Education Meeting, Seattle, Washington – 7 November 2013.

The views expressed in this article are those of the authors and do not necessarily reflect those of the Department of the Navy, Department of Defense, Department of Veterans Affairs, or any other U.S. Government Agency.

CAPT Sean R Maloney MC USN is a service member and employee of the U.S. Government. This work was prepared as part of his official duties. Title 17, USC, 105 provides that “Copyright protection under this title in not available for any work of the U.S. Government”. Title 17, USC, 101 defines a U.S. Government work as “a work prepared by a military service member or employee of the U.S. Government as part of that person’s official duties”.

The Authors would like to thank the following for their medical input including initial review of the proposed study and assistance with the analysis of data for this paper:

1. LT Anthony Skrypek MSC, USN for assistance in quickly obtaining data to be analyzed from the Naval Hospital Camp Lejeune medical record data bases.

2. CDR Earl Frantz MC USN and CAPT Steve Blivin MC USN, Department of Family Medicine, Naval Hospital Camp Lejeune, N.C for their clinical input and review of the proposed chart review.

3. CAPT Todd McCune MC USN, for his clinical input, encouragement, and support at the DPC-E for this chart review.
